# Type 2 diabetes susceptibility genes on mouse chromosome 11 under high sucrose environment

**DOI:** 10.1186/s12863-020-00888-6

**Published:** 2020-07-23

**Authors:** Misato Kobayashi, Hironori Ueda, Naru Babaya, Michiko Itoi-Babaya, Shinsuke Noso, Tomomi Fujisawa, Fumihiko Horio, Hiroshi Ikegami

**Affiliations:** 1 Department of Geriatric Medicine, Osaka University Graduate School of Medicine, Osaka, Japan; 2 Department of Animal Sciences, Graduate School of Bioagricultural Sciences, Nagoya University, Nagoya, Japan; 3 Department of Molecular Endocrinology, Osaka University Graduate School of Medicine, Osaka, Japan; 4 Health Care Center, KSC branch, Kwansei Gakuin University, Sanda, Hyogo Japan; 5 Department of Endocrinology, Metabolism and Diabetes, Kindai University Faculty of Medicine, 337-2 Ohno-higashi, Osaka-sayama, Osaka, 589-8511 Japan; 6 Health Care Center, Rinku General Medical Center, Osaka, Japan; 7 Sakai City Medical Center, Osaka, Japan

**Keywords:** Congenic strain, Consomic strain, Insulin resistance, Insulin secretion, NSY mice, Sucrose, Susceptibility gene, Type 2 diabetes

## Abstract

**Background:**

Both genetic and environmental factors contribute to type 2 diabetes development. We used consomic mice established from an animal type 2 diabetes model to identify susceptibility genes that contribute to type 2 diabetes development under specific environments. We previously established consomic strains (C3H-Chr 11^NSY^ and C3H-Chr 14^NSY^) that possess diabetogenic Chr 11 or 14 of the Nagoya-Shibata-Yasuda (NSY) mouse, an animal model of spontaneous type 2 diabetes, in the genetic background of C3H mice. To search genes contribute to type 2 diabetes under specific environment, we first investigated whether sucrose administration deteriorates type 2 diabetes-related traits in the consomic strains. We dissected loci on Chr 11 by establishing congenic strains possessing different segments of NSY-derived Chr 11 under sucrose administration.

**Results:**

In C3H-Chr 11^NSY^ mice, sucrose administration for 10 weeks deteriorated hyperglycemia, insulin resistance, and impaired insulin secretion, which is comparable to NSY mice with sucrose. In C3H-Chr 14^NSY^ mice, sucrose administration induced glucose intolerance, but not insulin resistance and impaired insulin secretion. To dissect the gene(s) existing on Chr 11 for sucrose-induced type 2 diabetes, we constructed four novel congenic strains (R1, R2, R3, and R4) with different segments of NSY-derived Chr 11 in C3H mice. R2 mice showed marked glucose intolerance and impaired insulin secretion comparable to C3H-Chr 11^NSY^ mice. R3 and R4 mice also showed impaired insulin secretion. R4 mice showed significant decreases in white adipose tissue, which is in the opposite direction from parental C3H-Chr 11^NSY^ and NSY mice. None of the four congenic strains showed insulin resistance.

**Conclusions:**

Genes on mouse Chr 11 could explain glucose intolerance, impaired insulin secretion, insulin resistance in NSY mice under sucrose administration. Congenic mapping with high sucrose environment localized susceptibility genes for type 2 diabetes associated with impaired insulin secretion in the middle segment (26.0–63.4 Mb) of Chr 11. Gene(s) that decrease white adipose tissue were mapped to the distal segment of Chr 11. The identification of diabetogenic gene on Chr 11 in the future study will facilitate precision medicine in type 2 diabetes by controlling specific environments in targeted subjects with susceptible genotypes.

## Background

Type 2 diabetes is a common disease driven by complex interactions between genetic and environmental factors [1]. The global prevalence of type 2 diabetes has been increasing at a rapid rate over the last several decades. Energy-rich diets containing high levels of fat and refined carbohydrates are thought to be the most significant environmental factors that contribute to obesity and type 2 diabetes development [2]. Thus, an understanding of genetic and environmental factors contributing to type 2 diabetes is crucial for developing preventative strategies and novel therapies. Human genome-wide association studies (GWAS) have identified susceptibility genes and pathways important in type 2 diabetes development. However, it is often difficult to clarify the genes involved in the development of diabetes under specific environment in humans due to genetic background heterogeneity and difficulty in controlling environmental factors. Therefore, inbred animal diabetes models are invaluable for elucidating the genetic factors in human type 2 diabetes development under specific environment.

The Nagoya-Shibata-Yasuda (NSY) mouse is an inbred mouse model of spontaneous type 2 diabetes with mild obesity [3]. The NSY mouse shows both impaired insulin secretion and insulin resistance caused by multiple genes. Two quantitative trait loci (QTLs) for glucose intolerance have been mapped on mouse chromosomes (Chr) 11 (*Nidd1n*) and 14 (*Nidd2n*) [4]. The effects of these QTLs on Chr 11 and 14 were confirmed using each consomic mouse, a strain possessing either Chr 11 or 14 of diabetic NSY mice in the genetic background of control C3H mice [5].

Diabetes development in NSY mice was affected by environmental factors like in human type 2 diabetes [6]. In NSY mice, a high-sucrose diet resulted in increased body weight gain, liver steatosis, and higher glucose intolerance compared to a high-fat diet [7]. We hypothesized that sucrose administration deteriorates the diabetes-related phenotypes of each consomic mouse possessing diabetogenic Chr 11 or 14 of the NSY mice, providing that diabetogenic genes on each chromosome are responsible for the deterioration of diabetes-related traits under the high sucrose environment. Here, we investigated whether sucrose administration deteriorates diabetes-related traits in the consomic strains. We also dissected the loci on Chr 11 by establishing novel congenic strains of mice under sucrose administration.

## Results

### Acceleration of diabetes and related phenotypes in consomic strains with sucrose administration

**Fig. 1:**
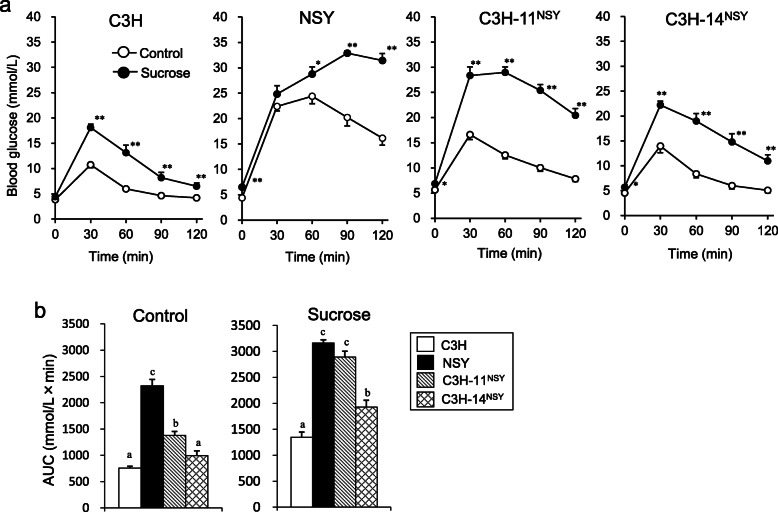
Glucose tolerance of C3H, NSY, C3H-Chr 11^NSY^, and C3H-Chr 14^NSY^. Mice were administered water (control) or 30% (w/v) sucrose solution (sucrose) from 4 weeks of age, and the phenotypes were studied at 12–14 weeks of age. **a** ipGTT. **b** gAUC during ipGTT. (Control group: C3H, *n* = 15; NSY, *n* = 9; C3H-Chr 11^NSY^, *n* = 15; C3H-14^NSY^, *n* = 6, Sucrose group: C3H, *n* = 6; NSY, *n* = 7; C3H-Chr 11^NSY^, *n* = 7; C3H-14^NSY^, *n* = 7). ^*^P < 0.05, ** *P* < 0.01 vs control group. ^a,b,c^ Means without common letters are significantly different by Tukey test (*P* < 0.05)

**Fig. 2:**
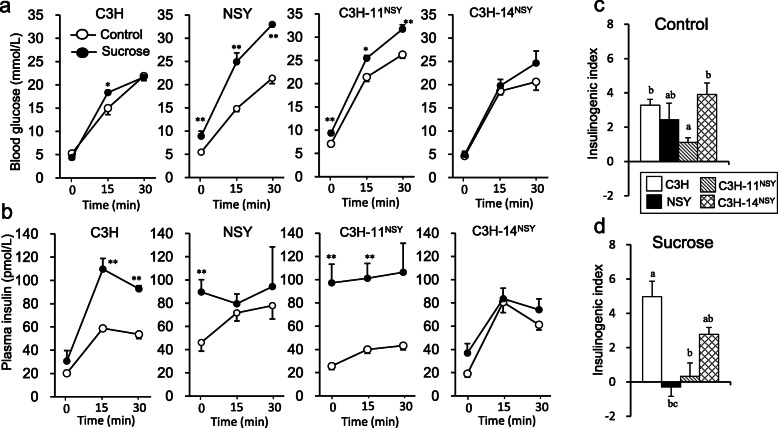
The assessment of insulin secretion of C3H, NSY, C3H-Chr 11^NSY^, and C3H-Chr 14^NSY^ mice administered water (control) or 30% sucrose solution (sucrose). **a** Blood glucose levels at 0, 15, and 30 min after glucose injection. **b** Plasma insulin levels at 0, 15, and 30 min after glucose injection. **c** Insulinogenic index in control group. **d** Insulinogenic index in sucrose group. (Control group: C3H, *n* = 12; NSY, *n* = 8; C3H-Chr 11^NSY^, *n* = 9; C3H-14^NSY^, *n* = 6, Sucrose group: C3H, *n* = 6; NSY, *n* = 7; C3H-Chr 11^NSY^, *n* = 5; C3H-14^NSY^, *n* = 7). ^*^P < 0.05, ** *P* < 0.01 vs control group. ^a,b,c^ Means without common letters are significantly different by Tukey test (*P* < 0.05)

**Fig. 3:**
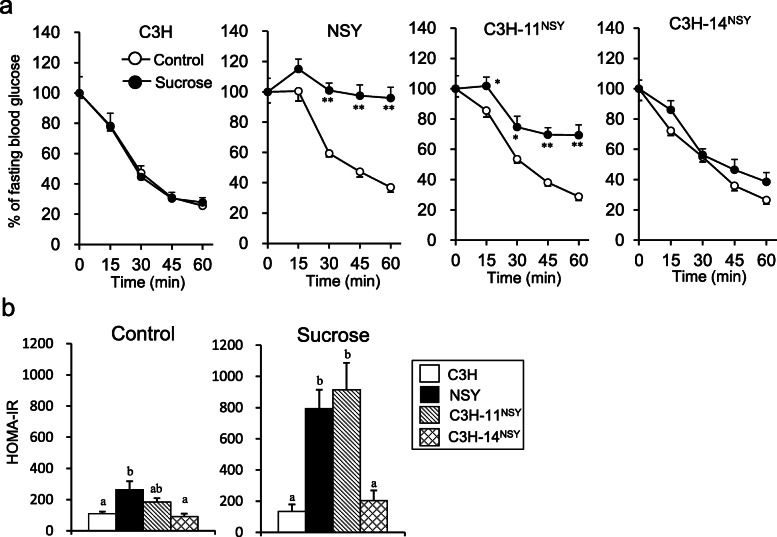
The assessment of insulin resistance of C3H, NSY, C3H-Chr 11^NSY^, and C3H-Chr 14^NSY^ mice administered water (control) or 30% sucrose solution (sucrose). **a** The percent of fasting blood glucose levels at 15, 30, 45, and 60 min after insulin injection. **b** HOMA-IR in control and sucrose groups. (Control group: C3H, *n* = 12; NSY, *n* = 8; C3H-Chr 11^NSY^, *n* = 9; C3H-14^NSY^, *n* = 6, Sucrose group: C3H, *n* = 6; NSY, *n* = 7; C3H-Chr 11^NSY^, *n* = 7; C3H-14^NSY^, *n* = 7). ^*^P < 0.05, ** *P* < 0.01 vs control group. ^a,b,^ Means without common letters are significantly different by Tukey test (*P* < 0.05)

To investigate the genomic regions involved in the deterioration of diabetes-related traits in NSY mice under the high sucrose environment, we administered sucrose to two consomic strains possessing diabetogenic Chr 11 and 14 of NSY mice on the genetic background of control C3H mice (C3H-Chr 11^NSY^ and C3H-Chr 14^NSY^, respectively). Sucrose administration deteriorated the glucose tolerance in NSY, C3H-Chr 11^NSY^, and C3H-Chr 14 ^NSY^ mice compared to those mice without sucrose (control groups) (Fig. 1a). C3H mice with sucrose administration also exhibited significantly higher blood glucose levels after a glucose injection compared to C3H mice without sucrose. The gAUC during ipGTT in C3H-Chr 11^NSY^ mice was significantly higher than C3H mice both in control and high sucrose environment, but it was much higher with high sucrose comparable to NSY mice (Fig. 1b). The gAUC in C3H-Chr 14 ^NSY^ was also significantly larger than C3H mice, but it was significantly smaller than NSY and C3H-Chr 11^NSY^ mice. To assess insulin secretion, blood glucose and plasma insulin levels at 0, 15, and 30 min after the glucose injection were measured (Fig. 2a and b). In control groups, plasma insulin levels at 15 and 30 min in all strains were higher than those at 0 min (Fig. 2b). Fasting plasma insulin levels of NSY and C3H-Chr 11^NSY^ mice in high sucrose groups were significantly higher than those without sucrose (control groups). Insulin secretion in response to glucose diminished in NSY and C3H-Chr 11^NSY^ mice with sucrose. In high sucrose groups, the insulinogenic indices in C3H-Chr 11^NSY^ mice were significantly lower than C3H mice, comparable to NSY mice (Fig. 2d). The glucose lowering effect of insulin, as assessed by decrease of blood glucose after an insulin injection, was significantly smaller in NSY and C3H-Chr 11^NSY^ mice with sucrose than those without sucrose (Fig. 3a). In contrast, the glucose lowering effect of insulin in C3H and C3H-Chr 14^NSY^ mice did not differ between control and sucrose groups. Under sucrose administration, HOMA-IR in C3H-Chr 11^NSY^ mice were significantly higher than C3H mice (Fig. 3b), comparable to NSY mice. Thus, under high sucrose administration, C3H-Chr 11^NSY^ mice showed hyperglycemia, impaired insulin secretion, and insulin resistance that were comparable to parental NSY mice, indicating that a gene or genes on Chr 11 of the NSY mice could explain these phenotypes in NSY mice under high sucrose environment (Figs. 1b, 2d, and 3b). In contrast, C3H-Chr 14^NSY^ mice showed no significant difference in insulinogenic index, and HOMA-IR from C3H mice under high sucrose administration (Figs. 2d and 3b), suggesting that Chr 14 did not contribute to the impaired insulin secretion and insulin resistance that were observed in NSY mice with sucrose.

**Fig. 4:**
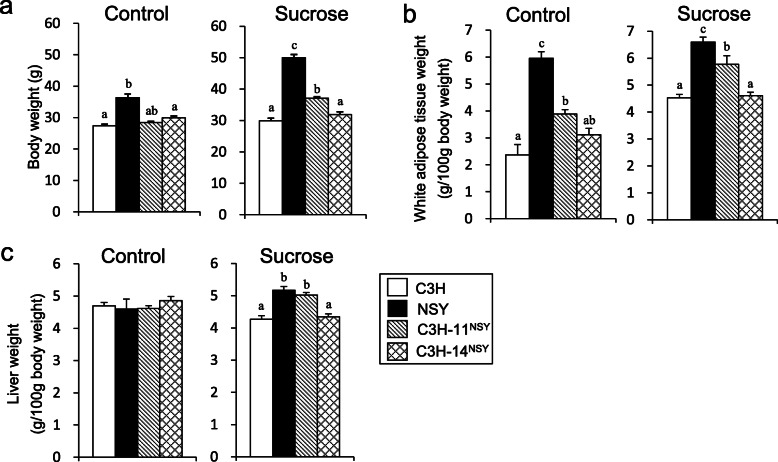
Phenotypic analyses of C3H, NSY, C3H-Chr 11^NSY^, and C3H-Chr 14^NSY^. Mice were administered water (control) or 30% (w/v) sucrose solution (sucrose) from 4 weeks of age, and the phenotypes were studied at 15 weeks of age. **a** Body weight. **b** White adipose tissue weight percent (sum of epididymal fat, retroperitoneal fat and mesenteric fat). **c** Liver weight percent. (Control group: C3H, *n* = 7; NSY, *n* = 7; C3H-Chr 11^NSY^, *n* = 13; C3H-14^NSY^, *n* = 6, Sucrose group: C3H, *n* = 6; NSY, *n* = 7; C3H-Chr 11^NSY^, *n* = 7; C3H-14^NSY^, *n* = 7). ^a,b,c^ Means without common letters are significantly different by Tukey test (*P* < 0.05)

In control groups, body weight and liver weight (g/100 g body weight) in C3H-Chr 11^NSY^ mice were comparable to C3H mice (Fig. 4a, c). Under sucrose administration, body weight, the percentages of white adipose tissue weights (sum of epididymal fat, retroperitoneal fat, and mesenteric fat; g/100 g body weight), and liver weight in C3H-Chr 11^NSY^ mice were significantly higher than C3H mice (Fig. 4). Liver triglyceride (TG) contents in NSY mice were significantly higher than C3H mice, and the liver TG content in C3H-Chr 11^NSY^ mice tended to be higher than C3H mice (Additional file 1).

### Establishment of new congenic strains

To dissect chromosomal regions involved in sucrose-induced deterioration of diabetes-related traits in C3H-Chr 11^NSY^ mice, we produced four congenic strains with different segments of NSY-derived Chr 11 (Additional file 2). The congenic strain R1 (C3H.NSY-[*D11Mit74-D11Mit229*]) possessed the proximal region (segment A) of NSY-Chr 11 from the centromere to the recombinant position between *D11Mit229* and *D11Mit163*. The congenic strain R2 (C3H.NSY-[*D11Mit74-D11Mit320*]) possessed the proximal and middle region (segment A, B and C) of NSY-Chr 11 from the centromere to the recombinant position between *D11Mit320* and *D11Mit94*. The congenic strain R3 (C3H.NSY-[*D11Mit242-D11Mit168*]) possessed the middle and distal region (segment C, D and E) of NSY-Chr 11 from the recombinant position between *D11Mit274* and *D11Mit242* to the telomere of Chr 11. The congenic strain R4 (C3H.NSY-[*D11Mit94-D11Mit168*]) possessed the distal region (segment E) of NSY-Chr 11 from the recombinant position between *D11Mit320* and *D11Mit94* to the telomere of Chr 11 (Additional file 2).

### Phenotypic analysis of four congenic strains, derived from C3H-Chr 11^NSY^, with sucrose administration

**Fig. 5:**
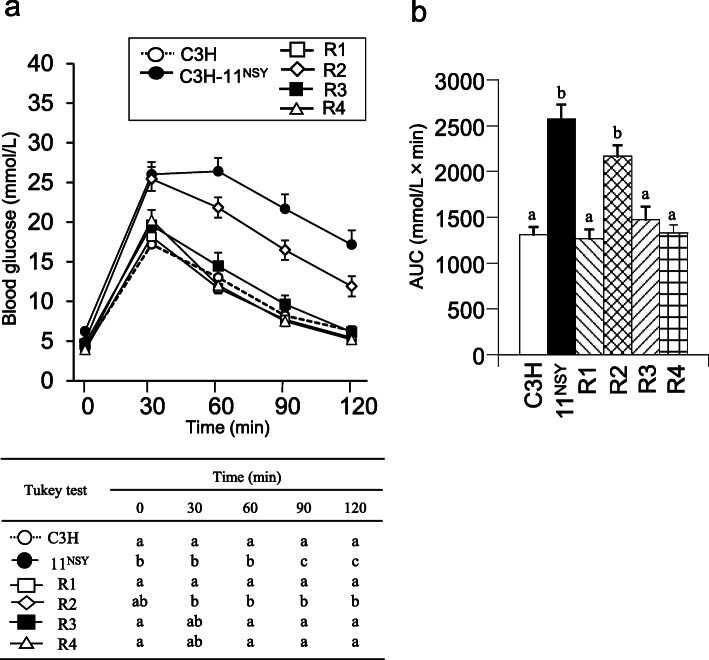
Glucose tolerance of C3H, C3H-Chr 11^NSY^, and four congenic strains (R1, R2, R3, and R4). Mice were administered 30% (w/v) sucrose solution from 4 weeks of age, and the phenotypes were studied at 12 weeks of age. **a** ipGTT. **)** gAUC during ipGTT. (C3H, *n* = 15; C3H-Chr 11^NSY^, *n* = 9; R1, *n* = 8; R2, *n* = 9; R3, *n* = 6; R4, *n* = 5). ^a,b,c^ Means without common letters are significantly different by Tukey test (*P* < 0.05)

**Fig. 6:**
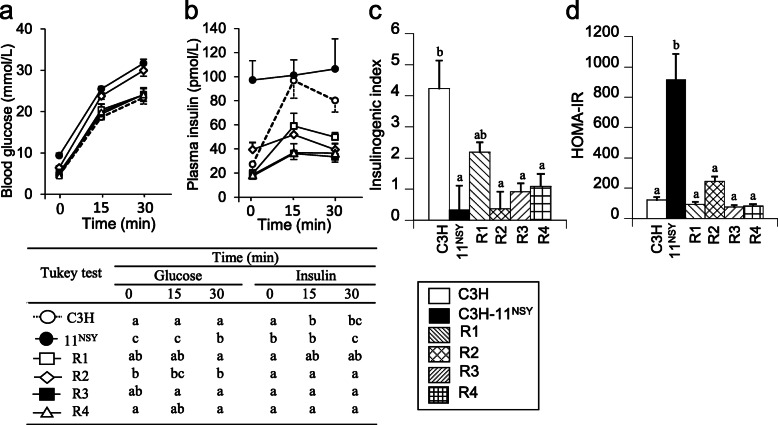
Insulin secretion and insulin sensitivity of C3H, C3H-Chr 11^NSY^, and four congenic strains (R1, R2, R3, and R4). Mice were administered 30% (w/v) sucrose solution from 4 weeks of age, and the phenotypes were studied at 13–14 weeks of age. **a** Blood glucose levels at 0, 15, and 30 min after glucose injection. **b** Plasma insulin levels at 0, 15, and 30 min after glucose injection. **c** Insulinogenic index. (C3H, *n* = 8; C3H-Chr 11^NSY^, *n* = 5; R1, *n* = 8; R2, *n* = 8; R3, *n* = 5; R4, *n* = 5). **d** HOMA-IR. (C3H, *n* = 8; C3H-Chr 11^NSY^, *n* = 5; R1, *n* = 8; R2, *n* = 8; R3, *n* = 5; R4, *n* = 5). **a-d** Data of C3H-Chr 11^NSY^ mice were same data used in Figs. 2 and 3. ^a,b,c^ Means without common letters are significantly different by Tukey test (*P* < 0.05)

Fasting glucose in R2 mice did not significantly differ from C3H-Chr 11^NSY^ mice, but it was significantly lower in R1, R3, and R4 mice compared to C3H-Chr 11^NSY^ mice (Fig. 5a). Glucose levels at all time points after a glucose challenge were significantly higher in R2 mice than C3H mice (Fig. 5a). Hyperglycemia in R2 mice, as assessed by gAUC during ipGTT, was comparable to C3H-Chr 11^NSY^ mice and significantly more severe than in C3H mice (Fig. 5b). Hyperglycemia did not differ significantly between R1, R3, and R4 mice and C3H mice (Fig. 5b). Plasma insulin levels at fasting did not differ between 4 congenic mice with sucrose and hyperinsulinemia observed in C3H-Chr 11^NSY^ mice were not observed in these congenic mice (Fig. 6b). Plasma insulin levels at 15 and 30 min after glucose injection in R2, R3, and R4 congenic mice were significantly lower than C3H mice (Fig. 6b). Therefore, insulinogenic indices in R2, R3, and R4 mice were significantly lower than C3H mice, but it did not differ significantly between R1 and C3H mice (Fig. 6c). HOMA-IR did not differ significantly between R1, R2, R3, and R4 mice and C3H mice (Fig. 6d), but it was significantly higher in C3H-Chr 11^NSY^ mice compared to C3H mice. These data suggest that glucose intolerance in R2 mice, which is as severe as C3H-Chr 11^NSY^ mice, is due to insulin secretion impairment in response to glucose rather than insulin resistance.

**Fig. 7:**
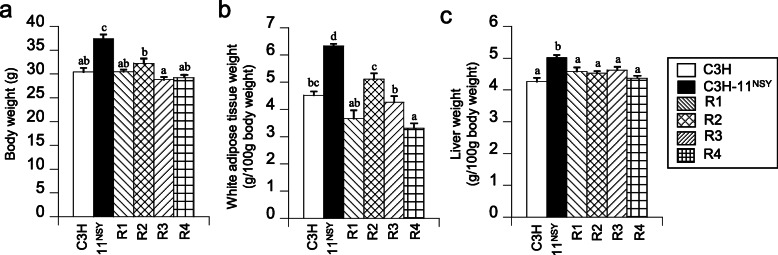
Phenotypic analyses of C3H, C3H-Chr 11^NSY^, and four congenic strains (R1, R2, R3, and R4). Mice were administered 30% (w/v) sucrose solution from 4 weeks of age, and the phenotypes were studied at 15 weeks of age. **a** Body weight. **b** White adipose tissue weight percentages (sum of epididymal fat, retroperitoneal fat, and mesenteric fat). **c** Liver weight percentages. (C3H, *n* = 6; C3H-Chr 11^NSY^, *n* = 4; R1, *n* = 6; R2, *n* = 7; R3, *n* = 8; R4, *n* = 5). ^a,b,c,d^ Means without common letters are significantly different by Tukey test (*P* < 0.05)

Body weights and white adipose tissue weights of four congenic strains did not differ from C3H mice, in contrast to significantly higher values observed in C3H-Chr 11^NSY^ mice (Fig. 7a, b). White adipose tissue weights in R4 mice were significantly lower than C3H mice, which was in the opposite direction from C3H-Chr 11 ^NSY^ mice with significantly higher values (Fig. 7b). The liver weight of four congenic strains did not differ from C3H mice (Fig. 7c).

## Discussion

**Fig. 8:**
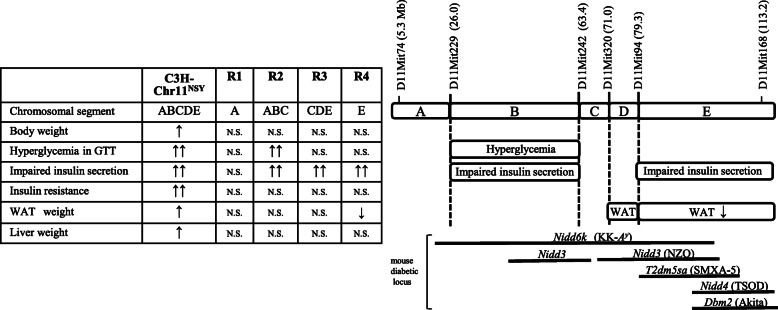
Summary of congenic phenotype and the chromosomal segments controlling the diabetes-related traits. ↑, in C3H-11^NSY^ mice, means significantly higher body weight and WAT weight than those in C3H mice, but not comparable to NSY mice. ↑↑, in C3H-11^NSY^ mice, means significantly impaired glucose tolerance and insulin secretion or higher liver weight than C3H, comparable to NSY mice. ↑↑, in R2 congenic strain, means significantly impaired glucose tolerance and impaired insulin secretion than C3H mice, comparable to C3H-11^NSY^ mice. ↑↑, in R3 and R4 congenic strains, means significantly impaired insulin secretion than C3H mice, comparable to C3H-11^NSY^ mice. ↓, in R4 congenic strain, means significantly lower tissue weight than C3H mice. N.S. means not significant. Black bars indicate the locations of reported mouse diabetic loci

This study demonstrated that mouse Chr 11 harbors a gene or genes involved in high sucrose-induced development of type 2 diabetes associated with impaired insulin secretion, insulin resistance, obesity, and fatty liver. Congenic mapping demonstrated the existence of genes for these traits in different Chr 11 segments. We found a gene or genes for hyperglycemia and impaired insulin secretion in the middle segment, one for decreased adiposity in the distal segment, and an interaction of multiple latent genes for obesity, adiposity, and insulin resistance (Fig. 8).

Epidemiological studies indicated that a high sucrose environment is a risk factor of obesity and type 2 diabetes in humans based on the association between high consumption of sugar sweetened beverages and increased obesity and type 2 diabetes incidence [8–11]. However, it is often difficult to clarify the genes involved in the development of diabetes under high sucrose environment in humans due to genetic background heterogeneity and difficulty in controlling environmental factors. Our previous study showed that NSY mice under sucrose administration for 12 weeks developed markedly impaired glucose tolerance, impaired insulin secretion, and insulin resistance associated with obesity [7]. This suggested that NSY mouse possessed genetic factors responsible for the development of these phenotypes under the high sucrose environment. Supporting the existence of the genetic factors, we previously reported the existence of susceptibility genes for type 2 diabetes on Chr 11 and 14 using C3H-Chr 11^NSY^ and C3H-Chr 14^NSY^ consomic mice, which possess diabetogenic Chr 11 and 14, respectively, in the NSY mouse in the control C3H mouse genetic background. C3H-Chr 11^NSY^ mice under a chow diet showed hyperglycemia, impaired insulin secretion, and insulin resistance without obesity, and C3H-Chr 14^NSY^ mice exhibited hyperglycemia and age-dependent insulin resistance with slight obesity [5]. However, diabetes-related phenotypes were not apparent in these consomic strains at a younger age. There was no insulin resistance in C3H-Chr 11^NSY^ and C3H-Chr 14^NSY^ mice and normal insulin secretion in response to glucose in C3H-Chr 14^NSY^ mice at 3 months of age. Here, sucrose administration clearly accelerated these phenotypes in C3H-Chr 11^NSY^ consomic strain. These data provide evidence for a gene or genes involved in the development of diabetes under high sucrose environment in NSY mice; genes on Chr 11 contribute to deterioration of hyperglycemia, impaired insulin secretion, and insulin resistance under high sucrose environment. Interestingly, C3H-Chr 11^NSY^ mice with sucrose administration showed increased body weight, fat accumulation, and increase in liver weight as early as 3 months of age, but this was never observed with a normal chow diet up to 12 months of age [5]. Thus, genes on Chr 11 under high sucrose environment contributed to either phenotype acceleration (early development and marked degree of hyperglycemia, impaired insulin secretion, and insulin resistance) or new phenotype development (obesity, adiposity, and fatty liver).

Here, we used congenic strains possessing different segments of Chr 11 from NSY mice in the control C3H genetic background to dissect the region of Chr 11 affecting hyperglycemia, impaired insulin secretion, and insulin resistance. R2, but not the R1, R3, or R4 congenic strains, carried the genes affecting hyperglycemia under sucrose administration (Fig. 5a, b), indicating that a locus for hyperglycemia is located in the middle region of Chr 11 encompassing from *D11Mit229* to *D11Mit242* (26.0–63.4 Mb; segment B in Additional file 2 and Fig. 8). Impaired insulin section was shown in congenic strains except for R1, indicating that multiple genes causing impaired insulin secretion existed on Chr 11 (Fig. 6c). Impaired insulin secretion in R2, but not in R1, indicates that a locus for this phenotype is located in the middle region of Chr 11 (segment B and C in Fig. 8). R3 and R4 congenic strains also show impaired insulin secretion, indicating that another locus for this phenotype is located in the distal region of Chr 11 (segment E in Fig. 8). A similar degree of impaired insulin secretion in R3 and R4 excludes segments C and D for this phenotype, localizing a locus for impaired insulin secretion mentioned above in R2 to the segment B (Fig. 8), encompassing *D11Mit229* to *D11Mit242* (26.0–63.4 Mb). This region is the same as the region for hyperglycemia. These data together with no significant differences in insulin resistance between four congenic strains and C3H mice suggest that segment B harbors a gene or genes for hyperglycemia caused by impaired insulin secretion, but not by insulin resistance under high sucrose environment.

Although a high sucrose environment is a risk factor for obesity and type 2 diabetes in humans [8–11], it is often difficult to clarify whether the high sucrose environment directly increases type 2 diabetes or if the effect is secondary to the effect on obesity and adiposity. A recent meta-analysis suggested high sugar sweetened beverage consumption is associated with a greater incidence of type 2 diabetes independent of adiposity [12]. Here, marked hyperglycemia and markedly impaired insulin secretion without changes in obesity or adiposity were shown in the R2 congenic strain (Fig. 8), indicating that a gene located in segment B on Chr 11 promote type 2 diabetes independent of obesity and adiposity under high sucrose environment. Identification of this gene will provide important information on molecular mechanisms of beta-cell function under high sucrose environment, and this can support the development of effective methods for type 2 diabetes prevention and treatment.

Insulin resistance and obesity observed in consomic C3H-11^NSY^ possessing whole Chr 11 was not observed in any of the congenic strains possessing each segment of Chr 11 (Figs. 6d, 7a). This suggested that multiple genes that slightly or latently affect these traits are located on Chr 11, and a combination of multiple latent genes causes insulin resistance and obesity observed in the Chr 11 consomic strain. Increased adiposity observed in C3H-Chr 11^NSY^ mice was not observed in any congenic strains. To our surprise, we observed a decrease, rather than an increase, in adiposity in the R4 strain. This indicated that the gene which decreases adiposity is located in the distal region of Chr 11 (segment E) (Fig. 8). Increased adiposity in C3H-Chr 11^NSY^ consomic mice possessing whole Chr 11 and decreased adiposity in R4 possessing segment E (Fig. 8) together with no changes in adiposity in R1, R2, and R3 mice suggest that the middle region of Chr 11 (segment D; 71.0–79.3 Mb) harbors a gene or genes that increase adiposity. The effect of this gene is masked by the effect of a gene on distal Chr 11 (segment E in Fig. 8) that decreases adiposity, leading to no changes in R3 mouse adiposity. These data indicate the importance of consomic and congenic mapping in the dissection of gene-gene interactions in complex traits like adiposity and obesity.

Loci for type 2 diabetes have previously been mapped in the segments B, D, and E on Chr 11 using QTL analyses in other mouse type 2 diabetes models. A locus for hyperglycemia in GTT was mapped to segment B-D in KK-*A*^*y*^ mice (*Nidd6k*) [13], a locus for serum insulin was mapped to segment B-E in NZO (*Nidd3*) [14], and loci for hyperglycemia in GTT were mapped to segment E in SMXA-5 *(T2dm5sa)* [15], TSOD *(Nidd4)* [16], and Akita mouse (*Dbm2*) [17] (Fig. 8). In the NZO mouse, the phosphatidylcholine transfer protein (*Pctp*) gene (90.0 Mb) has been shown to control insulin sensitivity [18, 19]. Genes responsible for impaired insulin secretion and decreased adiposity have not been identified in other mouse models.

To translate our results from mouse Chr 11 to humans, we used the human GWAS catalog to collect SNPs associated with type 2 diabetes in the human chromosomal region syntenic to mouse Chr 11 (Additional file 3). Among GWAS identified genes in humans, the *GCK* gene (glucokinase) [20, 21] is well known as a causative gene for maturity-onset diabetes of the young (MODY) 2. In addition, the mutation in the *HNF1B* (HNF1 homeobox B, *Tcf2*) [22, 23] has been identified as the cause of MODY5. We previously reported sequence analyses of *Gck* [5] and *Hnf1b* (*Tcf2*) [4, 24, 25], which showed allelic variation between NSY and control C3H mice. The *Gck* gene (5.9 Mb) is located in the R1 and R2 congenic region (segment A in Fig. 8), and the *Hnf1b* gene (83.9 Mb) is located in the R3 and R4 congenic region (segment E in Fig. 8 and Additional file 3). Although R1 congenic mice possess the *Gck* gene from NSY mice, R1 did not show impaired insulin secretion. This indicates that the *Gck* gene is unlikely to be a responsible for impaired insulin secretion in a high sucrose environment. *Hnf1b* may be a candidate gene for impaired insulin secretion observed in R3 and R4 congenic strains (segment E in Fig. 8 and Additional file 3). Based on human GWAS, SGCD, which is a component of the sarcoglycan complex, and JADE2, which acts as an E3 ubiquitin ligase, were suggested as candidate genes for impaired insulin secretion and hyperglycemia in the R2 congenic region (segment B, Additional file 3). We previously reported sequence analyses of other candidate genes for type 2 diabetes on Chr 11 including glucose transporter 4 [26], thioredoxin [25], and nucleoredoxin [27]. Coding sequences of these gene did not have allelic variantion between NSY and C3H mice, suggesting that amino acid substitution in these genes was unlikely to be responsible for type 2 diabetes susceptibility.

## Conclusions

We demonstrated that Chr 11 harbors the genes involved in sucrose-induced deterioration of type 2 diabetes-related traits in NSY mice. Congenic mapping with a high sucrose environment localized susceptibility genes for type 2 diabetes associated with impaired insulin secretion in the middle and distal segments on Chr 11. A gene or genes that decrease white adipose tissue were mapped to the distal segment of Chr 11. The identification of diabetogenic gene on Chr 11 in the future study will facilitate precision medicine in type 2 diabetes by controlling specific environment in targeted subjects with susceptible genotypes.

## Methods

### Animals

Male NSY/Osa (NSY) mice and C3H/HeNCrj (C3H) mice were maintained in the animal facilities of Osaka University Graduate School of Medicine with a 12-h light/dark cycle (lights-on/off at 6:00/18:00 clock time) in an air-conditioned room (22–25 °C). Two consomic mouse strains (C3H-Chr 11^NSY^ and C3H-Chr 14^NSY^) were previously established using the speed congenic method [5]. Congenic lines were produced as previously described [28] by selecting males that possessed the genomic region of interest on Chr 11 using 18 microsatellite markers as shown in Additional file 2. New congenic lines were maintained by brother-sister mating. Mice were maintained under specific pathogen-free conditions in the animal facilities of Osaka University Graduate School of Medicine. Male mice were used for all experiments. All mice had free access to 30% sucrose solution (sucrose group) or water (control group) and a standard diet (CRF-1: Oriental Yeast, Tokyo, Japan). Mice were housed in PC7115HT cages (189 mm × 297 mm × 128 mm; Allentown Inc., New Jersey, USA),with four or fewer mice per cage. From 4 weeks of age, sucrose group mice were administered 30% (w/v) sucrose solution for 11 weeks as previously described [7]. The experimental designs were approved by Osaka University Graduate School of Medicine Committee on Animal Welfare (Approval number: 030038–444).

### Phenotypic analyses

The intraperitoneal glucose tolerance test (ipGTT) was performed using the following protocol at 8 weeks of sucrose solution administration (12 weeks of age). After 18 h of fasting (from 16:00 to 10:00), blood samples were collected from the tail vein (fasting blood glucose sample). 20% glucose solution was injected intraperitoneally (2 g glucose/kg body weight). Blood samples were collected 30, 60, 90, and 120 min after the injection. Blood glucose concentration was measured using a glucose oxidase method with Glutest E (Kyoto Daiichi Kagaku, Kyoto, Japan). The area under the glucose concentration curve (gAUC) was calculated according to the trapezoid rule from the glucose measurements at fasting, 30, 60, 90, and 120 min. Insulin secretion in response to glucose was assessed by ipGTT (2 g glucose/kg body weight) in overnight-fasted mice at 10 weeks of sucrose solution administration (14 weeks of age). Blood glucose and plasma insulin levels were measured at fasting, 15, and 30 min. Plasma insulin levels were measured using Mouse Insulin ELISA (Morinaga, Yokohama, Japan). The insulinogenic index was calculated as [incremental AUC of insulin (ΣΔiAUC)] divided by [incremental AUC of glucose (ΣΔgAUC)] as previously described [28]. ΣΔiAUC and ΣΔgAUC were calculated according to the trapezoid rule from the insulin and glucose levels. Insulin tolerance test (ITT) was performed by injecting human insulin (0.25 U/kg body weight) intraperitoneally in fasted mice for 18 h at 9 weeks of experimental period. Blood glucose level was measured at 0, 15, 30, 45, and 60 min. HOMA-IR, an indicator of insulin resistance, was calculated as previously described [29]. Anatomical phenotypes were studied at 11 weeks of experimental period (15 weeks of age). Mice were euthanized by cardiac puncture under anesthesia with intraperitoneal administration of pentobarbital sodium (200 mg/kg body weight, Dainippon, Osaka, Japan). Euthanasia was confirmed by cervical dislocation and tissues (epididymal, mesenteric, and retroperitoneal fat pads and liver) were dissected and weighed.

### Collection of human GWAS data in syntenic region of mouse Chr 11

The human chromosomal syntenic region of mouse Chr 11 was obtained from Ensembl (http://www.ensembl.org/). Human SNPs associated with “type 2 diabetes mellitus” were collected from the GWAS catalog (http://www.ebi.ac.uk/gwas/) in the syntenic regions.

### Statistical analysis

All results were expressed as mean ± standard error of the mean (SEM). To analyze the differences between control group and sucrose group, *F*-test were performed to examine the variances of control group and sucrose group in each strain were equal or unequal. When the variances of each group were equal, Student’s *t*-test was used to compare the means between control and sucrose groups in each strain. When the variances were unequal, Welch’s test was used. To analyze the differences among each strain, one-way ANOVA and subsequent Tukey test were used to compare the means. Differences with *p* < 0.05 were regarded as significant. All statistical analyses were also performed using Ekuseru-Toukei version 3.00 (Social Survey Research Information Co., Ltd.).

## Supplementary information

**Additional file 1.** Liver triglyceride content in consomic mice.

**Additional file 2.** The chromosomal construction of consomic strain (C3H-Chr 11^NSY^) and the four congenic strains.

**Additional file 3.** Type 2 diabetes associated SNPs in human GWAS identified on mouse chromosome 11.

## Data Availability

The datasets used and/or analyzed during the current study are available from the corresponding author on reasonable request.

## Abbreviations

Chr: Chromosome
ipGTT: Intraperitoneal glucose tolerance test
ITT: Insulin tolerance test
NSY: Nagoya-Shibata-Yasuda
QTL: Quantitative trait locus
HOMA-IR: Homeostasis model assessment-insulin resistance
WAT: White adipose tissue
