# Supplementary material for: Type 2 diabetes susceptibility genes on mouse chromosome 11 under high sucrose environment
Source: BMC Genet. 2020 Jul 23;21:81. doi: 10.1186/s12863-020-00888-6 (PMC7379357; doi:10.1186/s12863-020-00888-6)
Supplement: Supplementary file 1 — Additional file 1. Liver triglyceride content in consomic mice. [file 12863_2020_888_MOESM1_ESM.docx]

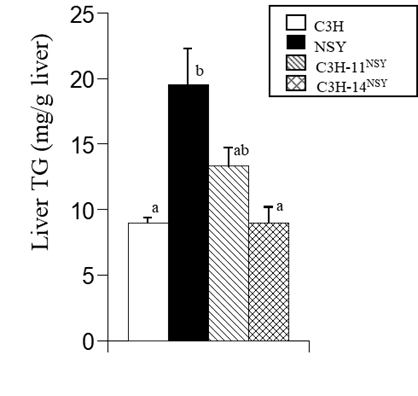


**Additional file 1:** Liver triglyceride content in consomic mice. Frozen livers were homogenized with chloroform: methanol (2:1), and liver lipids were extracted into organic solvents. A portion of this extract was dried, and the hepatic content of triglycerides was measured by the triglyceride E-test (Wako, Tokyo, Japan). (C3H, *n*=6; NSY, *n*=7; C3H-Chr 11^NSY^, *n*=6; C3H-14^NSY^, *n*=5).
