# Supplementary material for: Type 2 diabetes susceptibility genes on mouse chromosome 11 under high sucrose environment
Source: BMC Genet. 2020 Jul 23;21:81. doi: 10.1186/s12863-020-00888-6 (PMC7379357; doi:10.1186/s12863-020-00888-6)
Supplement: Supplementary file 2 — Additional file 2. The chromosomal construction of consomic strain (C3H-Chr 11NSY) and the four congenic strains. [file 12863_2020_888_MOESM2_ESM.docx]

**
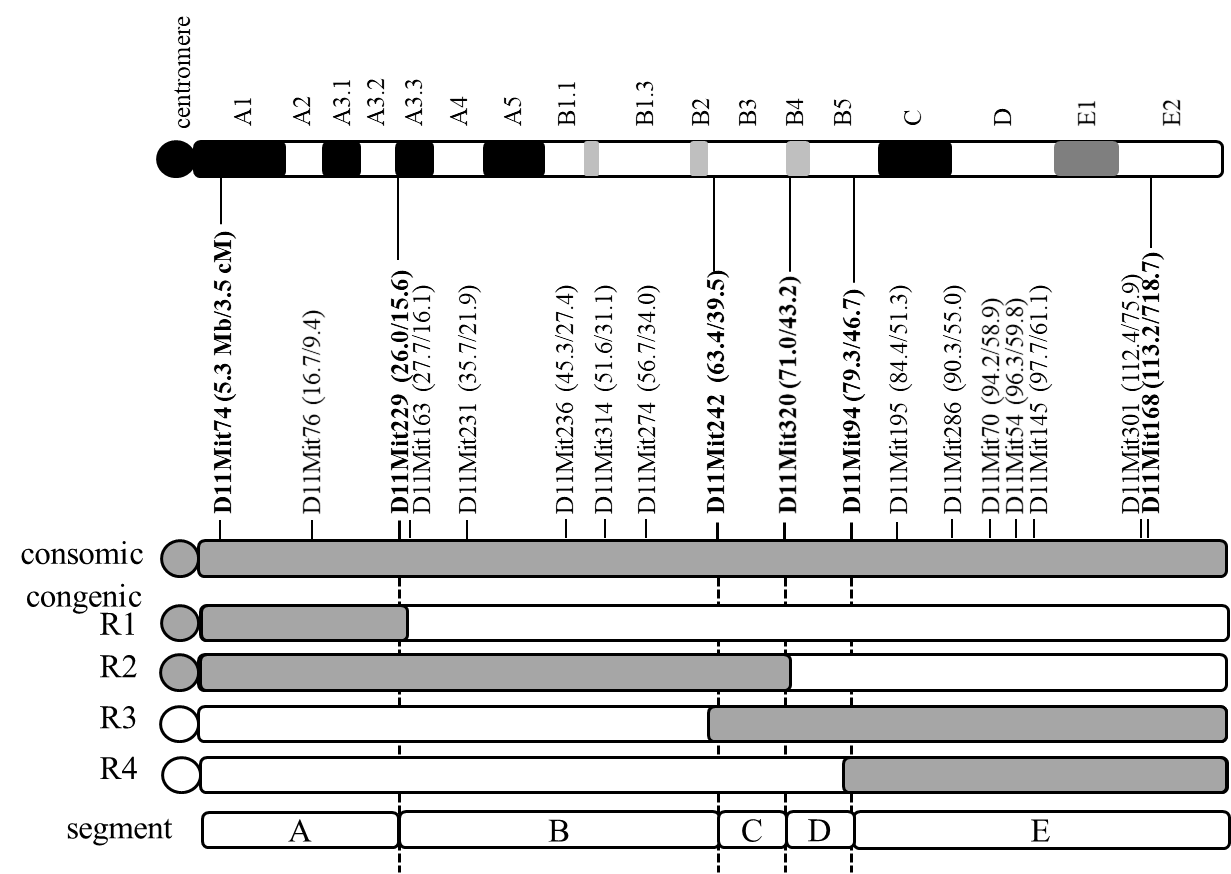
**

**Additional file 2:** The chromosomal construction of consomic strain (C3H-Chr 11^NSY^) and the four congenic strains. R1 (C3H.NSY-[*D11Mit74-D11Mit229*]), R2 (C3H.NSY-[*D11Mit74-D11Mit320*]), R3 (C3H.NSY-[*D11Mit242-D11Mit168*]) and R4 (C3H.NSY-[*D11Mit94-D11Mit168*]) carry NSY-derived susceptible regions in a C3H-derived resistance background. The gray bar shows the NSY strain-derived chromosomal region, and the white bar shows the C3H strain-derived chromosomal region. The positions (Mb and cM) of microsatellite markers were obtained from the Ensembl genome browser (NCBI m37).
